# Isolation and Functional Characterization of a Floral Repressor, *BcMAF1*, From Pak-choi (*Brassica rapa* ssp. *Chinensis*)

**DOI:** 10.3389/fpls.2018.00290

**Published:** 2018-03-06

**Authors:** Feiyi Huang, Tongkun Liu, Xilin Hou

**Affiliations:** State Key Laboratory of Crop Genetics and Germplasm Enhancement, Key Laboratory of Biology and Germplasm Enhancement of Horticultural Crops in East China, Ministry of Agriculture, College of Horticulture, Nanjing Agricultural University, Nanjing, China

**Keywords:** *BcAP3*, *BcMAF1*, *BcMAF2*, late flowering, Pak-choi (*Brassica rapa* ssp. *Chinensis*)

## Abstract

MADS-box genes form a large gene family in plants and are involved in multiple biological processes, such as flowering. However, the regulation mechanism of MADS-box genes in flowering remains unresolved, especially under short-term cold conditions. In the present study, we isolated *BcMAF1*, a Pak-choi (*Brassica rapa* ssp. *Chinensis*) *MADS AFFECTING FLOWERING* (*MAF*), as a floral repressor and functionally characterized *BcMAF1* in *Arabidopsis* and Pak-choi. Subcellular localization and sequence analysis indicated that BcMAF1 was a nuclear protein and contained a conserved MADS-box domain. Expression analysis revealed that *BcMAF1* had higher expression levels in leaves, stems, and petals, and could be induced by short-term cold conditions in Pak-choi. Overexpressing *BcMAF1* in *Arabidopsis* showed that *BcMAF1* had a negative function in regulating flowering, which was further confirmed by silencing endogenous *BcMAF1* in Pak-choi. In addition, qPCR results showed that *AtAP3* expression was reduced and *AtMAF2* expression was induced in *BcMAF1*-overexpressing *Arabidopsis*. Meanwhile, *BcAP3* transcript was up-regulated and *BcMAF2* transcript was down-regulated in *BcMAF1*-silencing Pak-choi. Yeast one-hybrid and dual luciferase transient assays showed that BcMAF1 could bind to the promoters of *BcAP3* and *BcMAF2*. These results indicated that *BcAP3* and *BcMAF2* might be the targets of BcMAF1. Taken together, our results suggested that *BcMAF1* could negatively regulate flowering by directly activating *BcMAF2* and repressing *BcAP3*.

## Introduction

The switch from vegetative to reproductive growth, which is called floral transition, is a vital developmental transition in flowering plants for reproductive success. The shoot apical meristem only produces leaf primordia during the vegetative phase, yet the shoot apical meristem can produce floral primordia after the transition. This process is regulated by multiple environmental and endogenous factors, such as temperature and daylength (Simpson and Dean, 2002). To maximize reproductive success, it is important to fulfill this transition at the correct time. Vernalization is a process that promotes plants to acquire flowering competence during long periods of winter cold and coordinating floral development with the seasons until winter has finished (Searle et al., 2006). The mechanisms by which plants regulate flowering time in the vernalization pathway, particularly during short-term cold conditions, and still coordinate floral development is a meaningful area of research.

MADS-box genes regulate multiple and important biological processes in plants, such as floral transition. MADS-box genes act in determining floral organ identity to control floral transition. Mutations of multiple MADS-box genes can change floral organs to leaves and overexpression of these genes can change leaves to floral organs. MADS-box proteins all have an MIKC structure and contain a highly conserved DNA-binding MADS domain in the N terminal, most of which can bind to a minimal consensus motif called the CArG box (Shore and Sharrocks, 1995). Some MADS-box genes are temperature responsive and regulate response to cold conditions. For example, *FLOWERING LOCUS C* (*FLC*) plays a key role in vernalization-induced flowering. In *Arabidopsis*, FLC functions as a floral repressor by directly repressing downstream genes, which promote flowering, such as *FLOWERING LOCUS T* (*FT*) and *SUPPRESSOR OF OVEREXPRESSION OF CONSTANS1* (*SOC1*) (Deng et al., 2011). The expression of *FLC* is stably reduced and triggered by vernalization on the epigenetic level and remains low even when returned to warm conditions. This repression can be “remembered” and reactivated only in the next generation, allowing rapid flowering in spring (Sheldon et al., 2000).

The genus *Brassica* has a close relationship with the model species *Arabidopsis*, both of which belong to the Brassicaceae family. *B. rapa* is a member of the genus *Brassica* whose subgenomes have evolved by genome fractionation from *Arabidopsis* (Wang et al., 2011). Flowering time is a key developmental trait and wide variation exists among *B. rapa* accessions. Several major flowering candidate genes, such as *FLC* and *FT*, have been identified in *B. rapa* based on previous studies of flowering time regulation. Six copies of *FT* have been mapped in *B. napus*, three of which were related to two major quantitative trait loci clusters for flowering time (Zhao et al., 2009). In addition, four *FLC* paralogs have been cloned in *B. rapa* (Schranz et al., 2002). The investigation of *FLC* expression and vernalization response in *B. napus* (Zou et al., 2012) and Chinese cabbage (Kim et al., 2007), together with co-localization of *FLC* paralogs with quantitative trait loci for flowering time in *B. rapa* (Lou et al., 2007; Xiao et al., 2013) suggests that the *Brassica FLC* genes and *Arabidopsis FLC* gene have similar functions. However, the cold-sensing mechanism of *FLC* is not clear and genetic manipulation of the vernalization trait in *B. rapa* has not been successfully studied.

*MADS AFFECTING FLOWERING*, which are *FLC*-related genes, also act as floral repressors in *Arabidopsis* (Ratcliffe et al., 2001). Although the functions of *MAFs* in *Arabidopsis* have been studied, there is no report on *MAF*s in Pak-choi. Pak-choi (*Brassica rapa* ssp. *Chinensis*), belonging to *B. rapa* family, is a major vegetable crop that is widely cultivated in Asia (Tian et al., 2004). The vernalization response shows difference among the different cultivars in Pak-choi. *Wuyueman* was used as the main material in the present study, which requires vernalization and flowers later than other cultivars. We isolated and functionally characterized *BcMAF1*, a Pak-choi *MAF* gene, as a floral repressor in *Arabidopsis* and Pak-choi. Expression profiles in different tissues and during the process of vernalization in Pak-choi were demonstrated. The results showed that *BcMAF1* was highly expressed in leaves, stems, and petals, and was induced by short-term cold conditions. Overexpressing *BcMAF1* in *Arabidopsis* caused late flowering and silencing endogenous *BcMAF1* in Pak-choi showed early flowering. Yeast one-hybrid assay, dual luciferase transient assay, and qPCR results showed that BcMAF1 could directly repress *BcAP3* and activate *BcMAF2.* In summary, the present study suggested that *BcMAF1* played a negative role in regulating flowering by directly repressing *BcAP3* and activating *BcMAF2* in Pak-choi.

## Materials and Methods

### Plant Materials

Plants of Pak-choi cultivars *Wuyueman* and *49caixin* were grown in a greenhouse under long day (LD) conditions (16 h light/8 h dark, 22°C/18°C). For the vernalization treatment, 1-month-old seedlings of Pak-choi cultivar *Wuyueman* were transferred to a new chamber exposure condition of 4°C for 5 weeks. Seedlings grown in the greenhouse without vernalization treatment were the control group. Leaves of vernalized and non-vernalized (control) seedlings were collected after treatment at 0, 1, 2, 3, 4, and 5 weeks; frozen immediately in liquid nitrogen; and stored at -80°C. To analyze the expression of *BcMAF1* in different tissues, the leaves, stems, roots, styles, stamens, petals and sepals of flowering Pak-choi cultivar *Wuyueman* were harvested and frozen in liquid nitrogen. Three biological replications were performed for each sample. The Pak-choi cultivar *49caixin* do not require a vernalization treatment and grow faster than other cultivars; therefore, it was only used for virus-induced gene silencing (VIGS).

*Arabidopsis* overexpressing *BcMAF1* seedlings were Col-0 ecotype background and grown in a greenhouse under LD conditions (16 h light/8 h dark, 22°C/18°C). Seeds of *35S:GFP* and *35S:BcMAF1-GFP* T_3_ lines were grown on Murashige and Skoog (MS) medium with 35 mg/L hygromycin for expression analysis of downstream genes. Whole seedlings were collected after 15 days for RNA extraction. Both Pak-choi and *Arabidopsis* samples used for qPCR were harvested at Zeitgeber time (ZT) 16 under LD conditions, in which *FT* might show relatively higher expression (Osnato et al., 2012). For extracting mesophyll protoplasts, 1-month-old *Arabidopsis* Col-0 seedlings were grown under short day (SD) conditions (8 h light/16 h dark photoperiod).

### Cloning and Sequence Analysis

To clone *BcMAF* genes, degenerate primers (5′-RATYGAGARCAARAGYAGTNGACAA-3′, Y = C/T, R = A/G, N = A/G/C/T) and 5′-Oligo(dT)20MN-3′(M = A/G/C, N = A/G/C/T) were first designed to amplify the conserved regions of the MAF orthologs based on sequence information from the *A. thaliana* MAF gene family in TAIR10^1^ and the Chinese cabbage *chiifu* genome in BRAD^2^ according to the previous report (Duan et al., 2015). Based on the sequencing results of PCR products from the conserved region of each MAF orthologs and the full length sequences of Chinese cabbage MAF orthologs (data not shown), we designed gene-specific primers (**Supplementary Table S1**) and performed 5′-and 3′-RACE (Smart RACE cDNA amplification kit; Clontech, Mountain View, CA, United States) to amplify the full-length cDNA sequences of *BcMAF*s in the cDNA library of Pak-choi cultivar *Wuyueman* leaves. Total RNA extraction, cDNA synthesis, and amplification by PCR were conducted using the same methods described in our previous report (Huang et al., 2016). The open reading frames (ORFs) of *BcMAF1* and *BcMAF2* (accession numbers: MG964044 and MG964045) were amplified by BcMAF1-S and BcMAF1-A, and BcMAF2-S and BcMAF2-A primers, respectively, and then inserted into the PMD18-T Vector (Takara, Beijing, China) before sequencing. The ORFs of *BcSOC1, BcFT1, BcFT2*, and *BcAP3* (accession numbers: MG964046, MG964047, MG964048, and MG964049) were also amplified using the same methods. The genomic sequences of *BcAP3* and *BcMAF2* were amplified by two pairs of primers, BcAP3-S and BcAP3-A, and BcMAF2-S and BcMAF2-A, respectively, from genomic DNA, which was isolated using the Plant Genomic DNA Kit (Tiangen, Beijing, China). The promoters of *BcAP3* and *BcMAF2* were obtained using corresponding SP1, SP2, and SP3 primers, which were designed based on the genomic sequences of *BcAP3* and *BcMAF2* by self-formed adaptor PCR with a KX Genome Walking Kit (Zoman Biotechnology, Beijing, China) according to the manufacturer’s instructions (Wang et al., 2007). The promoters were then amplified by two pairs of primers, Y1 and Y2, and Y3 and Y4. All primers used are listed in **Supplementary Table S1**. Multiple sequence alignment and phylogenetic analysis were performed based on the procedure outlined in our previous report (Huang et al., 2016). The phylogenetic tree was generated with full-length protein sequences using the Neighbor-Joining method by MEGA 6. Bootstrap values were estimated with 1000 replicates. The CArG boxes that existed in promoters were analyzed by the Softberry software program^3^.

### Subcellular Localization of *35S:BcMAF1-GFP* Protein

The protein coding region of *BcMAF1* without the termination codon was amplified by primers, O1 and O2, and then cloned into the pCambia1302 vector in fusion with the green fluorescent protein (GFP) under the CaMV35S promoter, generating a novel fusion vector (*35S:BcMAF1-GFP*). The empty vector (*35S:GFP*) was used as the control. *35S:BcMAF1-GFP* and *35S:GFP* plasmids were transformed into the *Agrobacterium tumefaciens* (strain GV3101) using the freeze-thaw method. The obtained *Ag. tumefaciens* were injected into tobacco leaves based on methods described previously (Zhang et al., 2012). Tobacco leaves were also stained with DAPI (nucleus specific dye) to confirm nuclear localization. After incubation at 25°C for 48 h, GFP in tobacco leaves was observed by confocal microscopy (Leica, TCS SP2, Wetzlar, Germany).

### Generation of *BcMAF1* Overexpressing Lines

The *35S:BcMAF1-GFP* construct was transformed into *Arabidopsis* Col-0 seedlings by the floral-dip method (Clough and Bent, 1998). Seeds of T_0_, T_1_, and T_2_ transgenic plants were selected on MS medium containing 35 mg/L hygromycin. To confirm the positive transgenic plants, PCR was used with a pair of specific primers (O1 and O2). The transgenic plants transformed with *35S:GFP* were used as the control. Western blot analysis was performed based on methods described in a previous study (Jie et al., 2017). The GFP fluorescence in transgenic plants was also detected based on the above method. Of the six independent transgenic lines selected, three T_3_ homozygous lines (#8, #16, and #23), confirmed by PCR, western blot, and GFP fluorescence observation, were used for all analyses. Flowering time was counted from sowing time to the opening time of the first flower. Total leaf number was counted until the time of bolting. Each measurement was calculated with 30 plants. Values were expressed as means ± standard deviation of mean (SEM). Analysis of variance was used for statistical analysis. Differences between lines were separated using the least significant difference test at *P* < 0.01.

### VIGS-Mediated Silencing of *BcMAF1* in Pak-choi

For VIGS assay, a 40 bp specific fragment of *BcMAF1* was derived from its coding sequence. The 40 bp specific fragment and its antisense version were synthesized by the GenScript company (China) to form the self-hybridized palindromic oligonucleotide. Then, the self-hybridized palindromic oligonucleotide was inserted into the *pTY-S* (*pTY*) vector of the turnip yellow mosaic virus-induced gene silencing (TYMV-VIGS) system to form the *BcMAF1*-silencing construct before sequencing (Pflieger et al., 2008). The *pTY* empty vector and the *pTY* carrying the self-hybridized palindromic oligonucleotide of *BcPDS* served as negative and positive controls, respectively. The sequences of oligonucleotides used for VIGS are listed in **Supplementary Table S2**. Two-week-old Pak-choi cultivar *49caixin* plants, which usually bolted at 8-weeks-old, were used for VIGS. The *pTY, pTY-BcPDS*, and *pTY-BcMAF1* plasmids (5 μg) coated onto gold particles were bombarded into plants by particle gun bombardment (Bio-Rad, PDS1000/He) based on the previous protocol with some modifications (Hamada et al., 2017). In each experiment, four plants were bombarded with each plasmid in three biological replicates. Three weeks later, leaves showing virus symptoms were sampled to analyze the levels of predicted downstream genes and silencing efficiency. *BcMAF1*-silencing Pak-choi plants were confirmed by qPCR. Two positive plants, *pTY-BcMAF1-1* and *pTY-BcMAF1-5*, were used for all experiments. Days from sowing to bolting time were counted.

### Expression Analysis in Pak-choi and *Arabidopsis*

qPCR analysis was performed based on methods described in our previous report (Huang et al., 2016). *AtACT* and *BcACT* were used as the internal controls in *Arabidopsis* and Pak-choi, respectively. To evaluate amplification specificity, melting curves were generated for each reaction. Three biological replicates were used for each experiment. The results were analyzed by the 2^-ΔΔC_t_^ method (Livak and Schmittgen, 2012). Primers for qPCR were designed by Premier 5 and are presented in **Supplementary Table S1**.

### Yeast One-Hybrid Assay

We performed the yeast one-hybrid assay with the Matchmaker^®^ Gold Yeast One-Hybrid System following the standard protocol (Clontech Laboratories Inc., Palo Alto, CA, United States). The promoter fragments of *BcMAF2* and *BcAP3* were cloned upstream of the reporter *AUR1-C* gene, which conferred resistance to Aureobasidin A (AbA), into the pAbAi vector at the *KpnI* and *XhoI* sites, respectively, and were then integrated into the yeast genome (strain Y_1_H GOLD). The information of the promoters of *BcMAF2* and *BcAP3* is shown in **Supplementary Table S3**. Yeast genomic PCR was used to verify bait strains by promoter-specific primers. The coding sequence of *BcMAF1* was inserted into pGADT7 with *EcoR*I and *SacI* restriction sites to generate the prey vector. After self-activation test, the prey vector was transformed into the yeast cells containing the promoter fragments on SD/-Leu medium. pGADT7 was used as the negative control. The survival colonies were picked and then spotted onto the SD/-Leu medium containing 300 ng/mL AbA at 30°C for 3 days.

### Transient Dual Luciferase Assay in *Arabidopsis* Protoplasts

To generate the reporter fusion construct, the putative promoter regions of *BcMAF2* and *BcAP3* were introduced into the pGreenII 0800-LUC vector using *Kpn*I and *Xho*I restriction sites, in which the Firefly luciferase gene (*FLUC*) was controlled by the above cloned promoters. Protoplasts were subsequently co-transfected with 20 μg of *35S:BcMAF1-GFP*, 20 μg of the recombinant pGreenII 0800-LUC vector, and 2 μg of Renilla luciferase (*RLUC*) reporter plasmid (as an internal control), and then incubated in darkness for 18 h. Protoplasts co-transfected with the recombinant pGreenII 0800-LUC plasmid and Renilla luciferase reporter plasmid were used as the negative control. Protoplasts isolation and transfection were performed following the described methods (Kang et al., 1998; Yoo et al., 2007) with some modifications. FLUC and RLUC activities were separately quantified by the Dual-Luciferase Reporter Assay System (Promega). The relative FLUC/RLUC activity was used to measure the promoter activity. All assays were repeated three times. Values are expressed as means ± SEM. Differences between treatments were separated using the least significant difference test at *P* < 0.01.

## Results

### Isolation and Expression Analysis of *BcMAF1*

**FIGURE 1:**
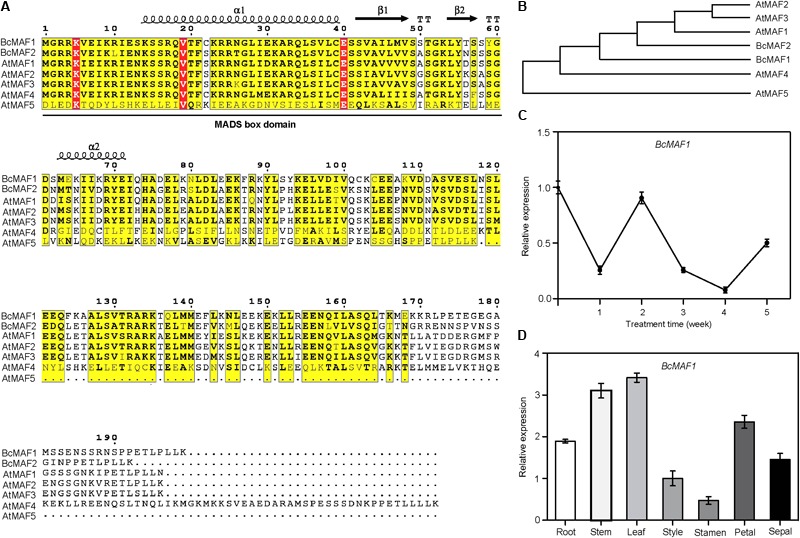
*BcMAF1* is a short-term cold response gene encoding an MADS protein. **(A)** Sequence alignment of BcMAF1, BcMAF2, and AtMAFs. Conserved and similar residues are boxed with red and yellow, respectively. The MADS-box domain is indicated by a straight line. **(B)** The phylogenic tree of BcMAF1, BcMAF2, and AtMAFs. The gene accession numbers are as follows: AtMAF1 (AT1G77080), AtMAF2 (AT5G65050), AtMAF3 (AT5G65060), AtMAF4 (AT5G65070), AtMAF1 (AT5G65080), BcMAF1 (MG964044), and BcMAF2 (MG964045). **(C)** The expression of *BcMAF1* during the process of vernalization in Pak-choi by qPCR. **(D)** The expression of *BcMAF1* in different tissues of Pak-choi by qPCR. Data shown are means ± SEM of three independent experiments.

We isolated two *MAF* genes in Pak-choi and named them as *BcMAF1* and *BcMAF2*. Multiple sequences alignment revealed that both BcMAF1 and BcMAF2 contained a typical MADS domain at the N-terminus (**Figure 1A**). Phylogenetic analysis of BcMAF1 and BcMAF2 with *Arabidopsis* MAFs were constructed (**Figure 1B**), which indicated that BcMAF1 and BcMAF2 showed greater similarity to AtMAF1, AtMAF2, and AtMAF3.

To determine whether the *BcMAF1* or *BcMAF2* expression was a response to vernalization in Pak-choi, we performed qPCR using total RNA from leaves of Pak-choi cultivar *Wuyueman* plants under cold treatment. Specifically, in the time-course analysis of treated samples, *BcMAF1* expression in leaves decreased after 1 week of cold treatment and reverted to its original value after 2 weeks, which was more rapid than the expression of *BcMAF2* (**Figure 1C** and **Supplementary Figure S1**). The abundance of *BcMAF1* transcript strongly decreased at 3 weeks, reached the minimum value at 4 weeks, and then increased. The results indicated that *BcMAF1* responded to vernalization and might play a more important role than *BcMAF2* in premature flowering under short-term cold conditions. We further analyzed the expression level of *BcMAF1* in different Pak-choi tissues. The highest *BcMAF1* transcript was found in leaves, followed by stems, petals, roots, sepals, styles, and stamens (**Figure 1D**).

### Subcellular Localization of BcMAF1 Protein

**FIGURE 2:**
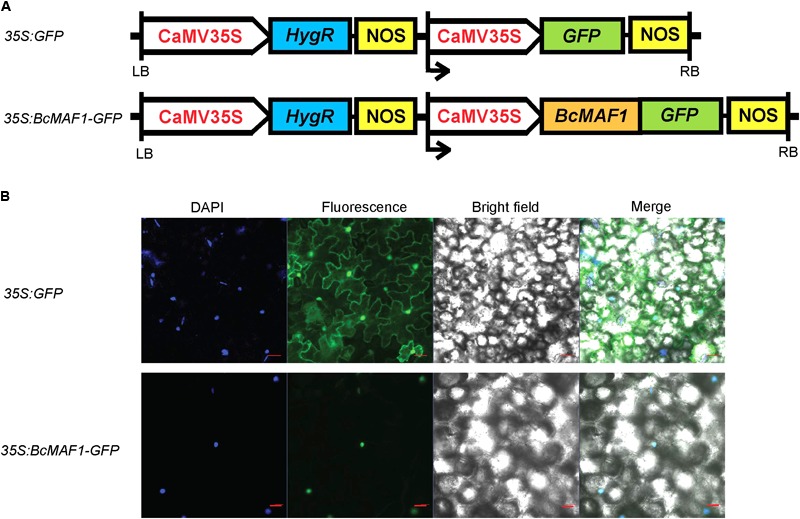
Subcellular localization of BcMAF1 protein. **(A)** *35S:GFP* and *35S:BcMAF1-GFP* construct. **(B)** Transient expression of *35S:GFP* (Scale bars = 50 μm) and *35S:BcMAF1-GFP* fusion protein (Scale bars = 20 μm) in tobacco leaves.

We detected the subcellular localization of *35S:BcMAF1-GFP* fusion (**Figure 2A**) by transiently overexpressing it in tobacco leaves using the *Agrobacterium* infiltration methodology. GFP fluorescence of the *35S:BcMAF1-GFP* fusion protein was only observed in the nucleus, which was also confirmed by DAPI staining. However, the fluorescence of *35S:GFP* was observed in both nucleus and cytoplasm (**Figure 2B**). This strongly suggested that BcMAF1 was targeted to the nucleus and might act as a transcription factor.

### Overexpression of *BcMAF1* in Transgenic *Arabidopsis* Caused Late Flowering

**FIGURE 3:**
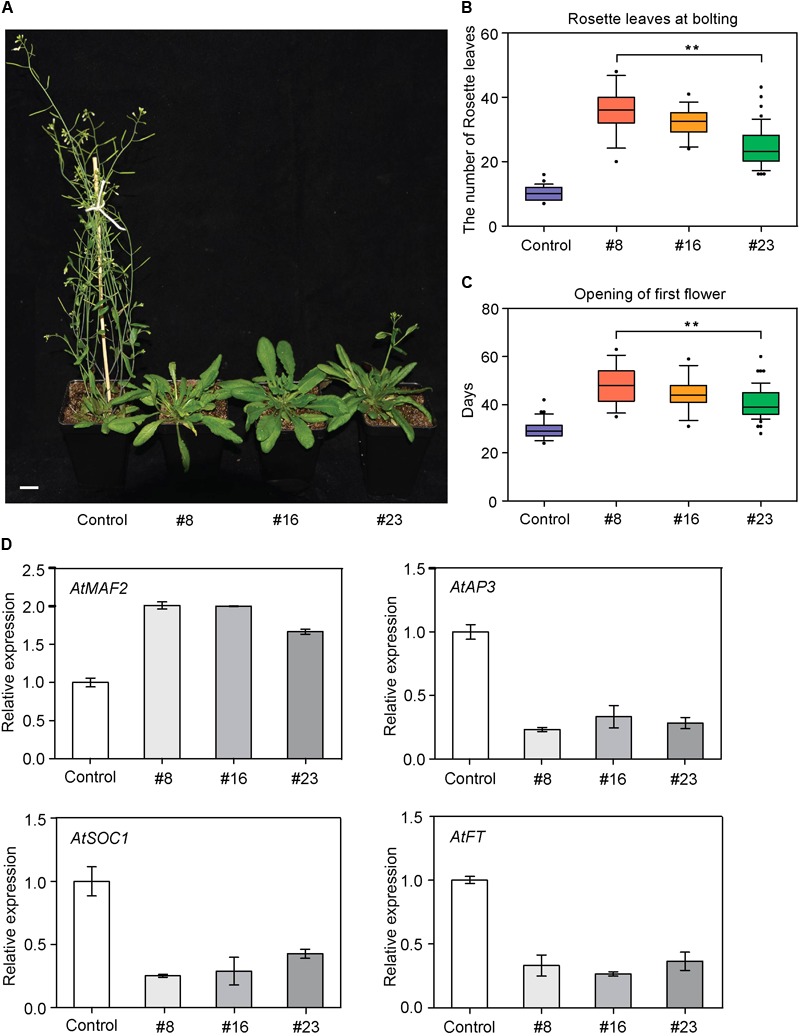
Overexpression of *BcMAF1* in *Arabidopsis.* **(A)** Late flowering phenotype of the transgenic plants overexpressed *BcMAF1*. The *35S:GFP* and *35S:BcMAF1-GFP#8, #16*, and *#23* plants in a chamber (LD, 16 h light/8 h dark photoperiod at 22°C/18°C). Scale bars = 1.5 cm. Rosette leaf number at bolting **(B)** Days of opening of first flower **(C)** in the *35S:GFP* and *35S:BcMAF1-GFP#8, #16*, and *#23* plants. Error bars represent standard deviation of the mean number of 30 plants for each line. ^∗∗^ indicate significant differences from the control (*P* < 0.01). **(D)** Expression analysis of predicted downstream genes in the *35S:GFP* and *35S:BcMAF1-GFP#8, #16*, and *#23* plants.

We first overexpressed *BcMAF1* in *Arabidopsis* Col-0 background to investigate its roles in flowering regulation. The PCR products of 597 bp (*BcMAF1* coding sequence without termination codon) were amplified in six T_3_ transgenic lines (#1, #4, #8, #11, #16, and #23), indicating that *BcMAF1* had been transformed and expressed in these six lines (**Supplementary Figure S2A**). The *BcMAF1*-GFP fusion protein of approximately 50 kDa was detected in four lines (#8, #11, #16, and #23), with the band of #11 weak (**Supplementary Figure S2B**). Meanwhile, the GFP fluorescence of three T_3_ lines (#8, #16, and #23) seedlings was also observed (**Supplementary Figure S2C**). These three T_3_ lines were used for further characterization. Transgenic plants that overexpressed *BcMAF1* showed markedly late flowering than control plants (**Figure 3A**). An increase in rosette leaves numbers (until the time of bolting) was observed in the three T_3_ lines (**Figure 3B**). Flowering time (days to opening of the first flower) was also delayed in the transgenic lines compared to the control line (**Figure 3C**). These results suggested that *BcMAF1* might function as a floral repressor.

To elucidate the molecular mechanism of *BcMAF1* in contributing to flowering repression, the expressions of four important genes involved in flowering were investigated. As shown in **Figure 3D**, the transcript level of *AtAP3*, which promoted flowering and was involved in specifying petal and stamen identities, decreased to less than 33% of the control in the transgenic lines. The transcript level of an important flowering repressor, *AtMAF2*, was also detected. The expression of *AtMAF2* showed an increase in the two-fold transcript of the control in transgenic lines. *AtSOC1* and *AtFT* (genes that promote flowering) expressions were approximately 57% lower in transgenic lines than in the control line. These results suggested that late flowering caused by overexpressing *BcMAF1* might be due to the inhibition of *AtAP3, AtSOC1*, and *AtFT*, and the activation of *AtMAF2* expression.

### Virus-Induced *BcMAF1* Silencing Caused Early Flowering

**FIGURE 4:**
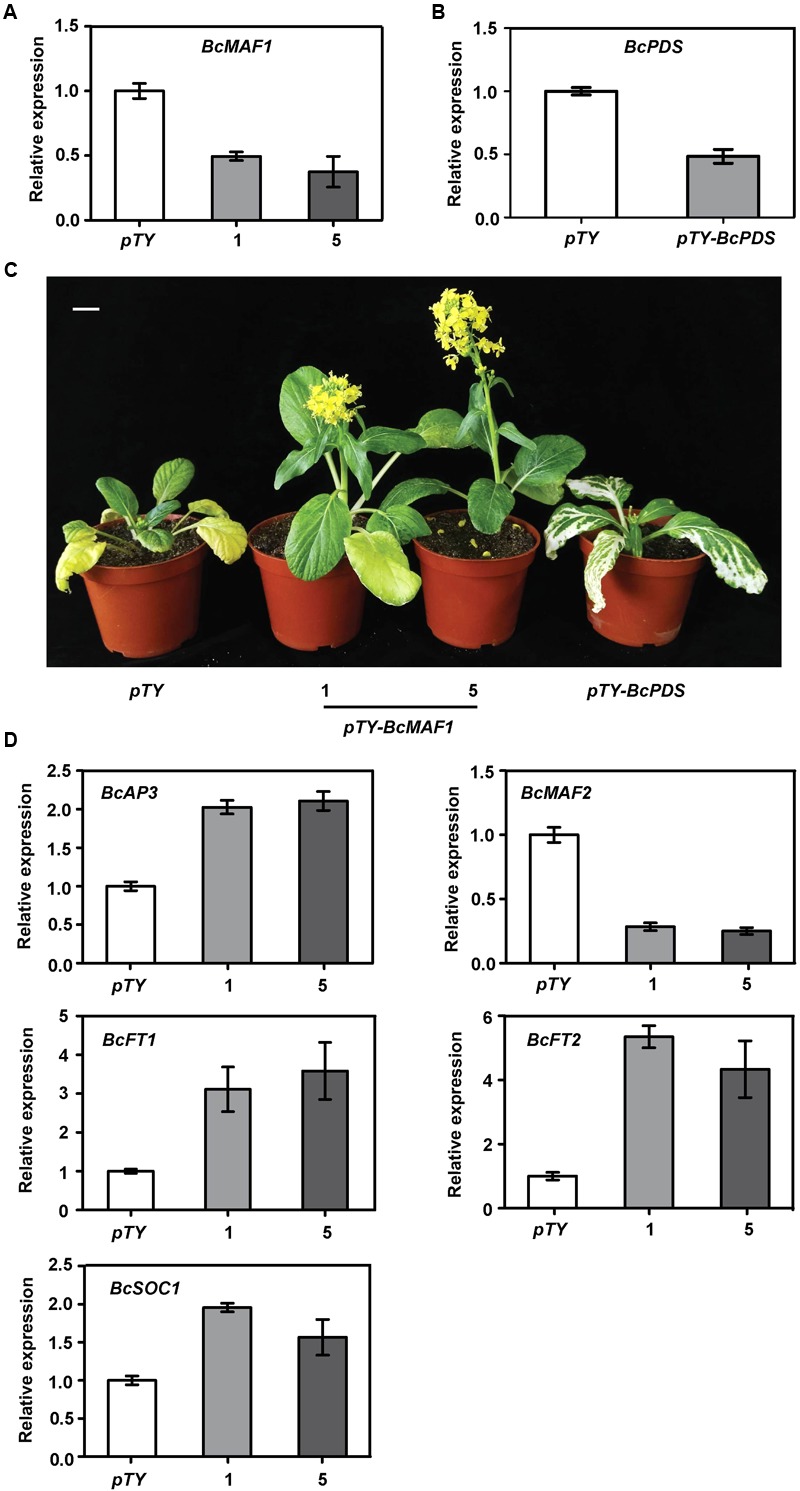
Silencing *BcMAF1* in Pak-choi. **(A)** Expression analysis of *BcMAF1* in Pak-choi seedlings bombarded with *pTY* and *pTY-BcMAF1* plasmid. **(B)** Expression analysis of *BcPDS* in Pak-choi seedlings bombarded with *pTY* and *pTY-BcPDS* plasmid. **(C)** Early flowering phenotype in Pak-choi seedlings silenced *BcMAF1*. Scale bars = 2.5 cm. **(D)** Expression analysis of predicted downstream genes in the *pTY, pTY-BcMAF1-1*, and *pTY-BcMAF1-5* plants.

To further functionally characterize the role of *BcMAF1* in Pak-choi flowering regulation, we silenced *BcMAF1* using TYMV-based VIGS. Three weeks after Pak-choi plants underwent particle gun bombardment, the photobleaching or mosaic leaf phenotype typical of Phytoene desaturase (PDS) deficiency or TYMV was observed on the upper leaves of *pTY-BcPDS, pTY-BcMAF1*, or *pTY* (control) plants, which indicated that TYMV-mediated gene silencing was effective in Pak-choi. Total RNA was extracted from the upper leaves of the positive plants. To confirm the efficiency of silencing, the expression levels of *BcMAF1* and *BcPDS* in the positive plants were evaluated with qPCR. *BcMAF1* expression was significantly reduced by at least 62% in *BcMAF1*-silencing plants, whereas *BcPDS* expression was significantly reduced by approximately 52% in *BcPDS*-silencing plants (**Figures 4A,B**). As expected, silencing *BcMAF1* accelerated bolting by 11–13 days (**Supplementary Table S4**) and promoted flowering (**Figure 4C**) in comparison to the control. We then detected the transcript levels of the predicted downstream genes. The levels of *BcAP3, BcFT1, BcFT2*, and *BcSOC1* were higher whereas the level of *BcMAF2* was lower in the *BcMAF1*-silencing plants than in the control plant (**Figure 4D**). This suggested that *BcAP3, BcMAF2, BcFT1, BcFT2*, and *BcSOC1* might participate in *BcMAF1*-mediated flowering regulation.

### Direct Binding of BcMAF1 to the Promoters of *BcMAF2* and *BcAP3*

**FIGURE 5:**
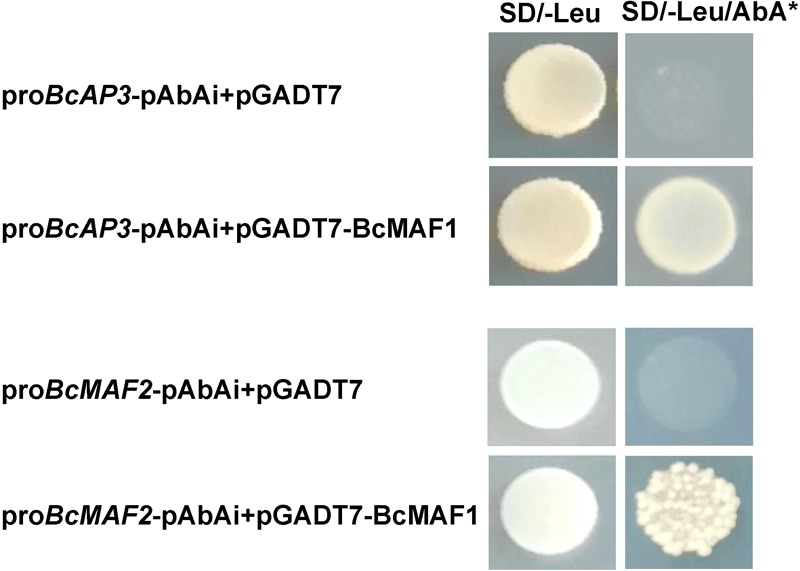
Binding activities of BcMAF1 protein with the promoters of *BcMAF2* and *BcAP3* detected by yeast one-hybrid assays. The yeast cells were grown on SD/-Leu medium plate supplemented with or without 300 ng/mL AbA.

A previous study showed that *SOC1* and *FT* were repressed by MAFs (Shen et al., 2014); therefore, we studied the relationships among *BcMAF1, BcAP3*, and *BcMAF2*. Some members of the MADS-box family can recognize and bind to the CArG box, a consensus sequence that has a core motif CC(A/T)_6_GG, in the promoters of their targets (Shore and Sharrocks, 1995). On account of *BcMAF1* belonging to the MADS-box family, we speculated that BcMAF1 could bind to promoters owning the CArG box. Transcription regulatory elements analysis indicated that *BcMAF2* and *BcAP3* promoters possessed at least one CArG box (**Supplementary Table S3**). Meanwhile, the expression levels of *AtMAF2* and *AtAP3* were significantly changed in *BcMAF1*-overexpressing *Arabidopsis* (**Figure 3D**), whereas the expression levels of *BcMAF2* and *BcAP3* were significantly changed in the *BcMAF1*-silencing Pak-choi (**Figure 4D**). This suggested that *BcMAF2* and *BcAP3* might be the targets of BcMAF1 in Pak-choi. To test and verify this hypothesis, we performed the yeast one-hybrid assay. Yeast cells harboring the promoter fragments of *BcAP3* or *BcMAF2* together with pGADT7-BcMAF1 were all obtained on SD/-Leu medium containing 300 ng/mL AbA, except for the control (**Figure 5**). The results implied that the BcMAF1 protein had DNA binding activity and could directly bind to the promoters of *BcMAF2* and *BcAP3*.

**FIGURE 6:**
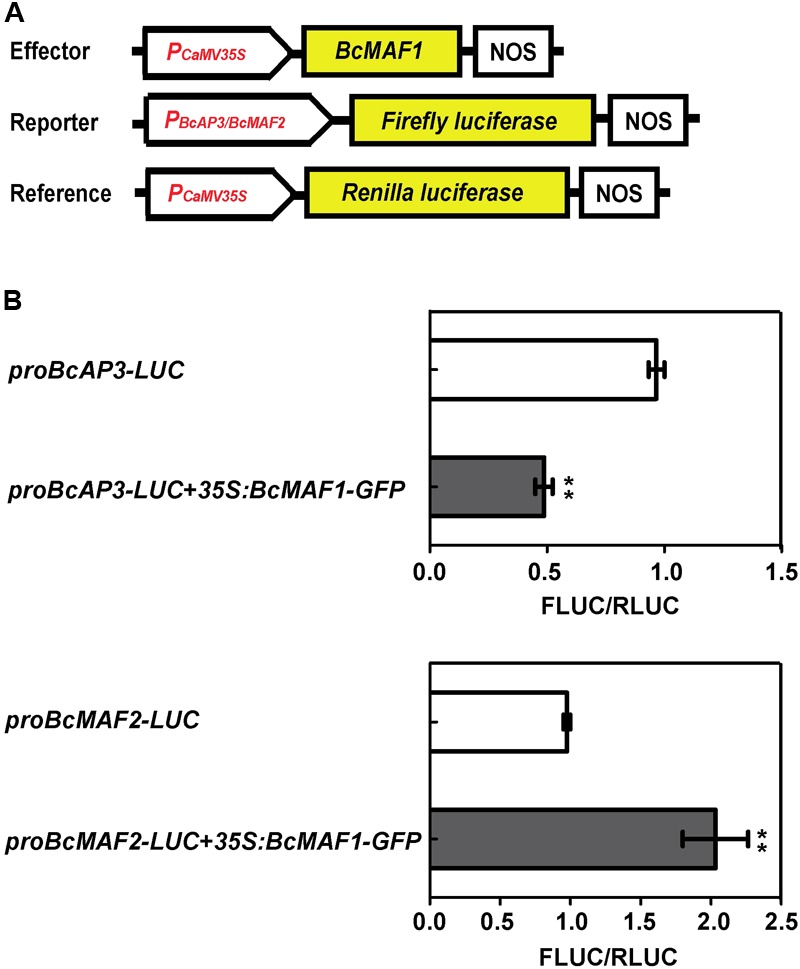
Activation or repression of *BcMAF2* or *BcAP3* promoter by overexpressing *BcMAF1* in the dual luciferase transient assay. **(A)** Diagram of the effector, reporter, and reference plasmids used in the dual luciferase transient assay. **(B)** The relative FLUC/RLUC activities of *BcMAF2* and *BcAP3* promoters activated and repressed by recombinant *35S:BcMAF1-GFP* in *Arabidopsis* protoplasts were detected by Dual-Luciferase^®^ Reporter Assay System. Bars represent the mean ± SEM. The relative FLUC/RLUC activity of the negative control was set at 1. ^∗∗^ indicate a significant difference at *P* < 0.01.

To further confirm the binding activity of BcMAF1 with pro*BcMAF2* and pro*BcAP3*, we conducted a transient expression experiment using the dual luciferase assay. When *35S:BcMAF1-GFP* was co-transfected with *proBcMAF2*-*LUC*, the value of FLUC/RLUC was approximately 2-fold higher than the control. In contrast, the value of FLUC/RLUC was approximately 50% lower than the control when *35S:BcMAF1-GFP* was co-transfected with *proBcAP3*-*LUC*. Together, our results proved that *BcMAF2* was activated by BcMAF1 directly binding to its promoter, yet *BcAP3* was inhibited by BcMAF1 (**Figure 6**).

## Discussion

In *Arabidopsis*, there are five MAF proteins that are highly related to FLC (with 53–87% identity), which show temperature-dependent changes in expression (De Bodt et al., 2003). They also repress floral transition and their expressions are regulated by vernalization (Ratcliffe et al., 2003; Sung et al., 2006). *FLC, MAF3*, and the other three *MAF* genes (*MAF1, MAF2*, and *MAF4*) can directly interact with each other and form complexes to regulate the genes related to floral transition, such as *FT* (Gu et al., 2013). However, the molecular mechanisms of *MAF*s in flowering regulation under short-term cold conditions are still not clear, especially in Pak-choi. In the present study, we successfully isolated a *MAF* gene and named it as *BcMAF1* from Pak-choi cultivar *Wuyueman*. We found that *BcMAF1* was mainly expressed in leaves, stems, and petals, and responded to vernalization. The sharply up-regulated expression of *BcMAF1* after 2 weeks of vernalization treatment suggested that *BcMAF1* might function in preventing premature flowering. Thus, we further investigated *BcMAF1* function by overexpressing it in *Arabidopsis* and silencing it in Pak-choi, as well as investigated the targets of BcMAF1 in Pak-choi.

Late flowering in *BcMAF1*-overexpressing *Arabidopsis* and early flowering in *BcMAF1*-silencing Pak-choi plants were found (**Figure 3A, Figure 4C**), which suggested that *BcMAF1* could repress flowering. MADS-box transcription factors could bind to the CArG box and two CArG boxes in the *BcAP3* promoter were also found (**Supplementary Table S3**). Additionally, *BcMAF1* was highly expressed in petals, whereas *AP3* was highly expressed in stamens and petals (Theissen et al., 2000). Therefore, we predicted that BcMAF1 might directly regulate *BcAP3* to control the floral transition time, which was in agreement with the down-regulation of *AtAP3* in transgenic *Arabidopsis* and up-regulation of *BcAP3* in *BcMAF1*-silencing Pak-choi (**Figures 3D, 4D**). Yeast one-hybrid and dual luciferase assays showed that BcMAF1 could directly bind to the *BcAP3* promoter (**Figures 5, 6**).

Floral organ identity is controlled by the combinatorial activity of five classes of floral homeotic genes in *Arabidopsis*, according to the ABCDE model (Theissen and Saedler, 2001). *AP3*, a B class gene, is a transcription factor of the MADS-box family and regulates many plant developmental processes, such as floral development and flowering (Irish and Kramer, 1998; Theissen et al., 2000). Mutations in *AP3* caused the conversions of petals to sepals and stamens to carpels in *Arabidopsis* (Bowman et al., 1989; Jack et al., 1992). Additionally, overexpressing poplar *AP3* in tobacco plants showed an earlier flowering phenotype (An et al., 2011). *AP3* had a positive feedback loop to maintain its own expression (Jack et al., 1992; Goto and Meyerowitz, 1994). AP3, PI, and AP1 could form a heterodimer and then bind to the CArG box *in vitro* (Honma and Goto, 2001). AP3 is also required for the regulation of female flower development by directly activating *ETR1* in the cucumber (Sun et al., 2016). Therefore, we further detected *AP1* and *ETR1* expressions in the transgenic *Arabidopsis* and *BcMAF1*-silencing Pak-choi. *AtAP1* and *AtETR1* transcript levels were lower in transgenic *Arabidopsis*, whereas *BcAP1-1, BcAP1-2*, and *BcETR1* transcript levels were higher in silencing Pak-choi (**Supplementary Figure S3**). These findings suggested that *BcMAF1* could also reduce *BcETR1, BcAP1-1*, and *BcAP1-2* by directly inhibiting *BcAP3* to regulate not only flowering time but also floral development in Pak-choi. The possible role of *BcMAF1* in floral development still requires further investigation.

Previous studies have shown that *MAF2*, which is encoded a floral repressor, can prevent premature vernalization under short periods of cold. The *maf2* mutant shows flower earlier than wild type after short periods of cold; however, it retains a normal vernalization response. This protection process is likely to be independent of *FLC* because *FLC* expression is not significantly decreased after short periods of cold (Ratcliffe et al., 2003). Thus, we predicted that *BcMAF2*, a *MAF2* homolog, might also play a key role in short-term cold conditions in Pak-choi, which was confirmed by qPCR (**Supplementary Figure S1**). The relationship between *BcMAF1* and *BcMAF2* was further investigated. Yeast one-hybrid and dual luciferase assays demonstrated that *BcMAF2* might be another target of BcMAF1 (**Figures 5, 6**). This explained the up-regulation of *AtMAF2* by overexpressing *BcMAF1* in transgenic *Arabidopsis* and the down-regulation of *BcMAF2* by silencing endogenous *BcMAF1* in Pak-choi (**Figures 3D, 4D**). Thus, we theorized that BcMAF1 could prevent premature flowering by directly activating *BcMAF2* in Pak-choi.

We also examined the temporal expression pattern of *BcMAF1* in Pak-choi under SD and LD conditions (**Supplementary Figure S4**). The abundance of *BcMAF1* was low in light conditions and increased 8 h before dusk, with a peak in transcript at dusk under SDs. However, the abundance of *BcMAF1* was not significantly changed under LD conditions. Meanwhile, *BcMAF1* expression was lower in Pak-choi plants grown under LD conditions than those grown under SD conditions, indicating that LDs may inhibit *BcMAF1* transcript to promote flowering. Overall, *BcMAF1* expression showed a circadian oscillation under SD conditions. Further research is required to study the role of *BcMAF1* under SD conditions.

Taken together, *BcMAF1* was a floral repressor and prevented premature flowering in Pak-choi. *BcMAF1* acted as a nuclear transcription factor and regulated the expressions of *BcAP3* and *BcMAF2* by directly binding to their promoters. This is the first study on the functional analysis of the *MAF* gene in Pak-choi flowering regulation. This research will help further clarify the regulatory mechanisms of flowering not only in Pak-choi but in other Brassicaceae species as well.

## Author Contributions

FH: Performed the experiments and wrote the paper. FH, TL, and XH: Manuscript revision and approval. XH: Contributed to the interpretation of the results and coordinated the study. All authors read and approved the final manuscript.

## Conflict of Interest Statement

The authors declare that the research was conducted in the absence of any commercial or financial relationships that could be construed as a potential conflict of interest.

## Footnotes

1 http://arabidopsis.org/index.jsp

2 http://brassicadb.org/brad/

3 http://www.softberry.com/

## Supplementary Material

FIGURE S1 The expression of *BcMAF2* during the process of vernalization in Pak-choi by qPCR.

FIGURE S2 Identification of T_3_ transgenic *Arabidopsis* plants. **(A)** PCR analysis of T_3_ transgenic *Arabidopsis* plants overexpressing empty vector (control) and *BcMAF1* (#1, #4, #8, #11, #16, and #23). The amplified fragments were the *BcMAF1* coding sequence without termination codon (597 bp). **(B)** Western blot detection of the *BcMAF1-GFP* fusion protein in the transgenic *Arabidopsis* plants. The fusion protein is approximately 50 kDa. The band of marker is 50 kDa. **(C)** GFP fluorescence observation of *35S:BcMAF1-GFP#8*, #*16*, and #*23* seedlings.

FIGURE S3 Expression analysis of predicted downstream genes of *BcAP3* in *BcMAF1*-overexpressing *Arabidopsis* **(A)** and *BcMAF1*-silencing Pak-choi **(B)**.

FIGURE S4 Changes in *BcMAF1* transcript in response to LD and SD were monitored in Pak-choi by qPCR. Data shown are means ± SEM of three independent experiments.

TABLE S1 Primers used in the paper.

TABLE S2 Sequences of oligonucleotides used for VIGS.

TABLE S3 Information of the promoters of *BcMAF2* and *BcAP3*.

TABLE S4 Days from sowing to the time of bolting in the silenced Pak-choi plants.
