# Supplementary figures and images for: Isolation and Functional Characterization of a Floral Repressor, BcMAF1, From Pak-choi (Brassica rapa ssp. Chinensis)
Source: Front Plant Sci. 2018 Mar 6;9:290. doi: 10.3389/fpls.2018.00290 (PMC5845726; doi:10.3389/fpls.2018.00290)

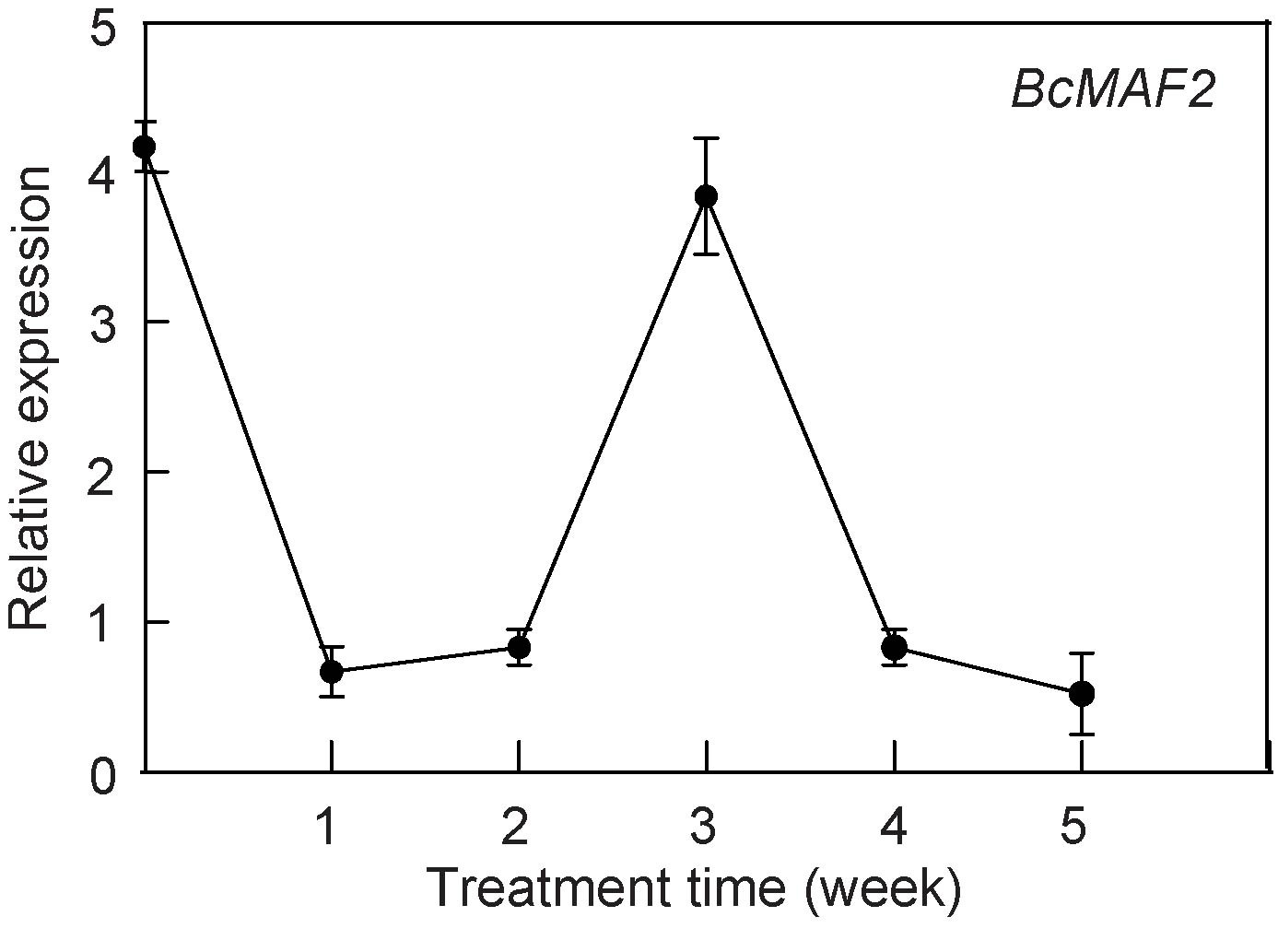

Supplement: FIGURE S1 — The expression of BcMAF2 during the process of vernalization in Pak-choi by qPCR. [file Image_1.JPEG]

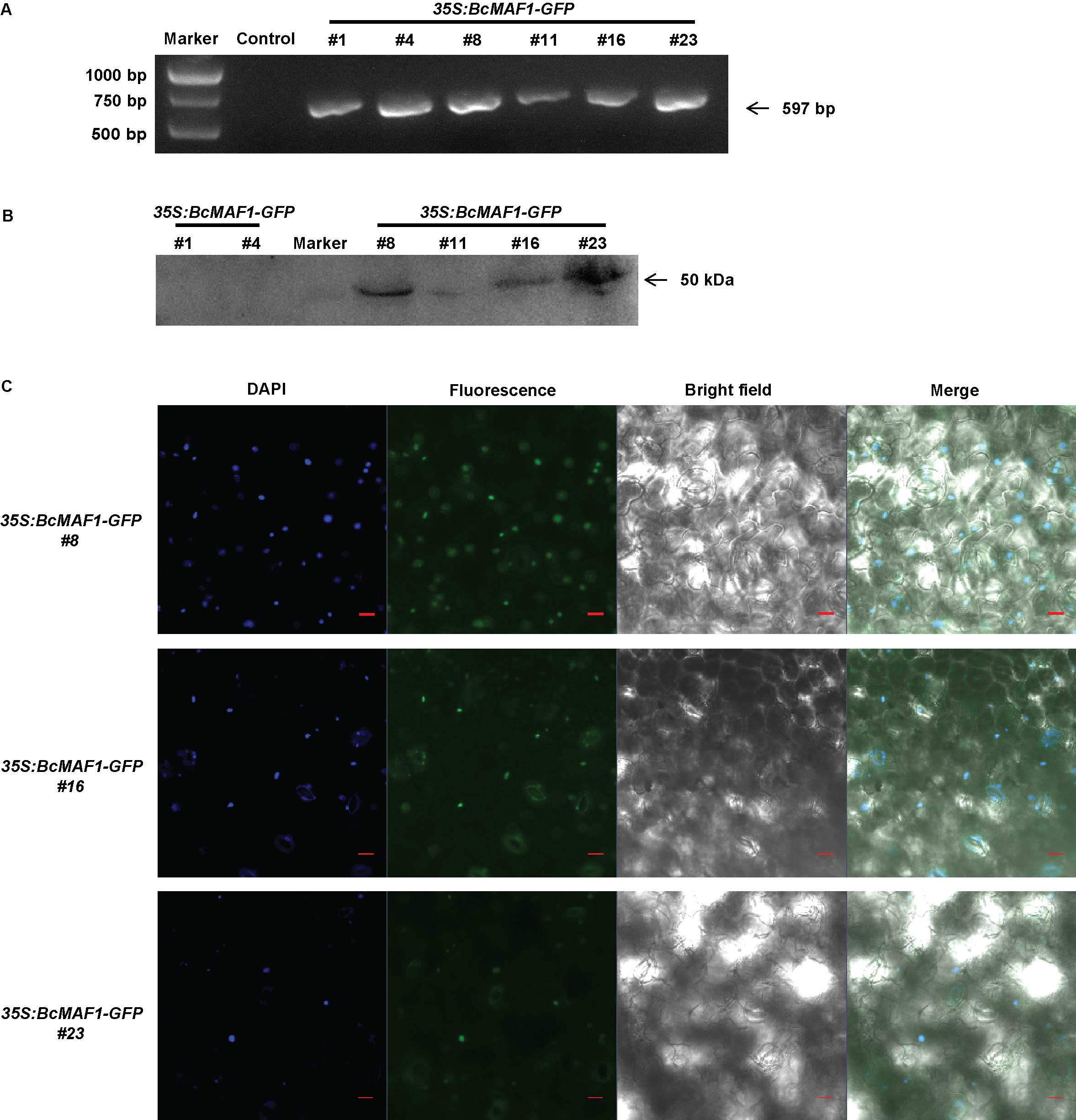

Supplement: FIGURE S2 — Identification of T3 transgenic Arabidopsis plants. (A) PCR analysis of T3 transgenic Arabidopsis plants overexpressing empty vector (control) and BcMAF1 (#1, #4, #8, #11, #16, and #23). The amplified fragments were the BcMAF1 coding sequence without termination codon (597 bp). (B) Western blot detection of the BcMAF1-GFP fusion protein in the transgenic Arabidopsis plants. The fusion protein is approximately 50 kDa. The band of marker is 50 kDa. (C) GFP fluorescence observation of 35S:BcMAF1-GFP#8, #16, and #23 seedlings. [file Image_2.JPEG]

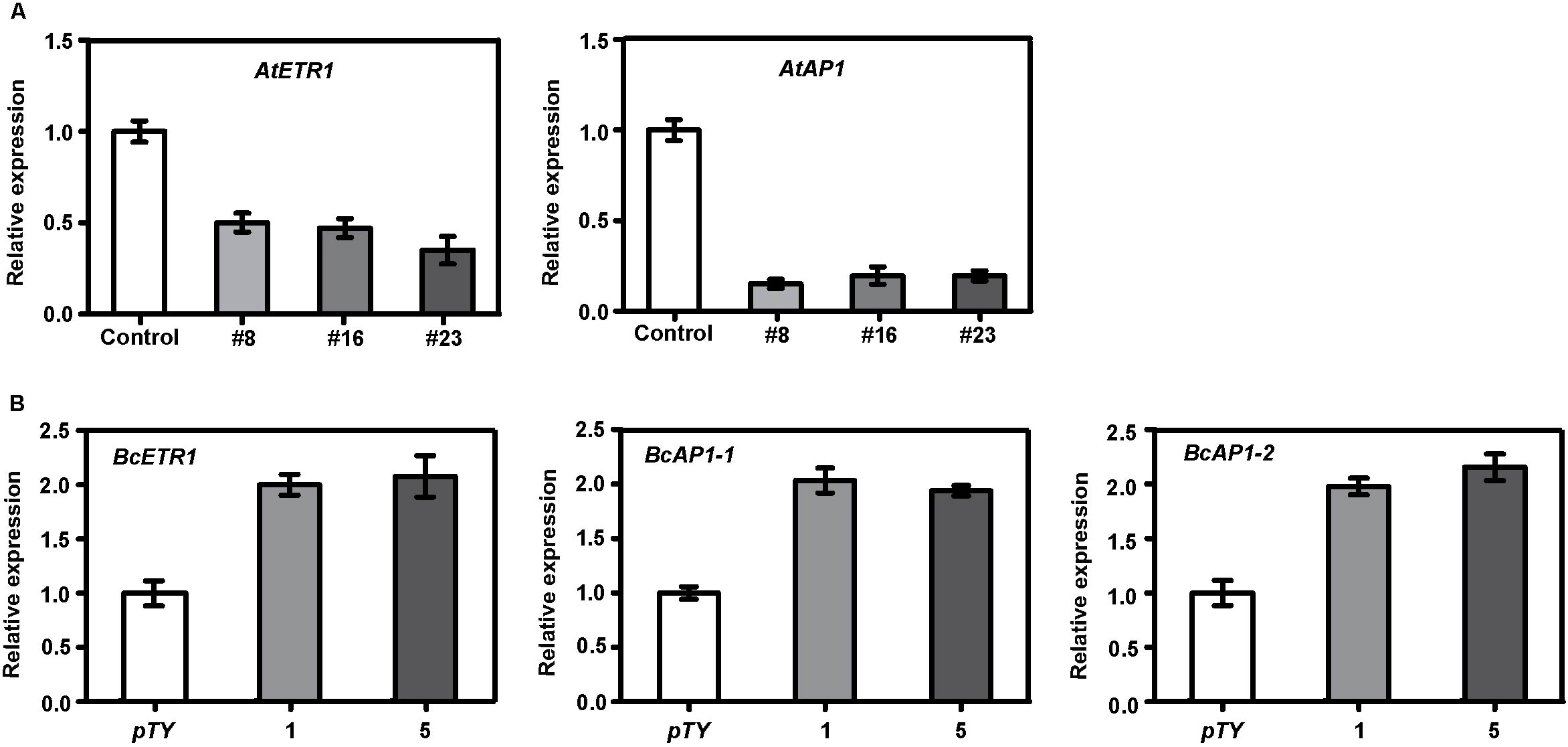

Supplement: FIGURE S3 — Expression analysis of predicted downstream genes of BcAP3 in BcMAF1-overexpressing Arabidopsis (A) and BcMAF1-silencing Pak-choi (B). [file Image_3.JPEG]

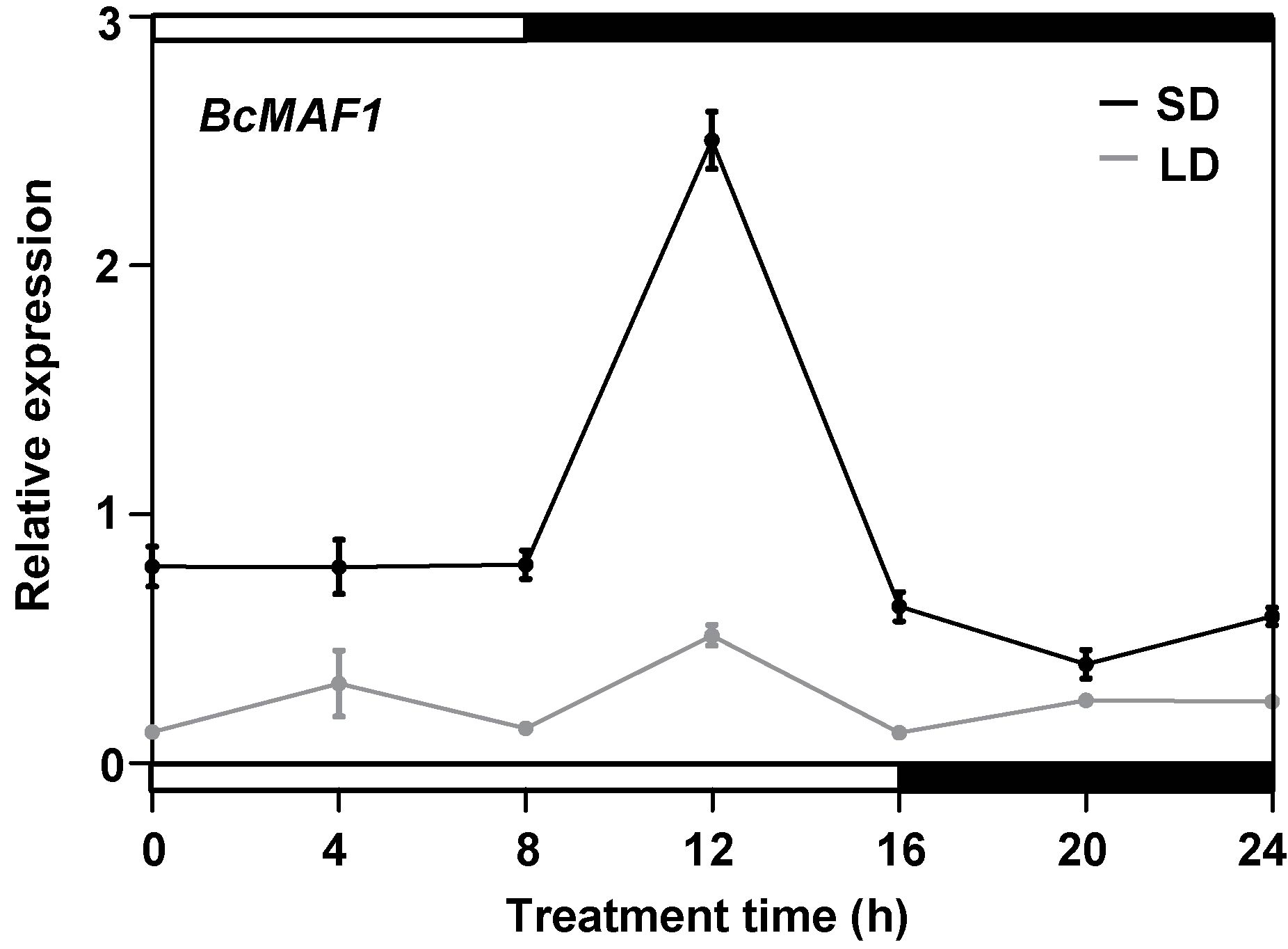

Supplement: FIGURE S4 — Changes in BcMAF1 transcript in response to LD and SD were monitored in Pak-choi by qPCR. Data shown are means ± SEM of three independent experiments. [file Image_4.JPEG]
